# The Lymphatic System, Lymphoedema, and Medical Curricula–Survey of Australian Medical Graduates

**DOI:** 10.3390/cancers14246219

**Published:** 2022-12-16

**Authors:** Natalie Kruger, Melanie L. Plinsinga, Rhian Noble-Jones, Neil Piller, Vaughan Keeley, Sandra C. Hayes

**Affiliations:** 1 Menzies Health Institute Queensland, Griffith University, Nathan, Brisbane, QLD 4111, Australia; 2 School of Health Sciences and Social Work, Griffith University, Brisbane, QLD 4111, Australia; 3 Physiotherapy Department, Royal Brisbane and Women’s Hospital, Nathan, Brisbane, QLD 4006, Australia; 4 Lymphoedema Clinical Network Wales, Swansea University, Swansea SA2 8PP, UK; 5 School of Medicine, Flinders University, Adelaide, SA 5042, Australia; 6 Lymphoedema Department, University Hospitals of Derby and Burton NHS Foundation Trust, Derby DE22 3NE, UK

**Keywords:** medical education, graduate physician, lymphatic system, lymphedema

## Abstract

**Simple Summary:**

Lymphoedema is a common medical condition, with early identification leading to more timely management and better outcomes. However, people with lymphoedema in Australia express frustration about the lack of knowledge of their condition among doctors and other healthcare professionals, resulting in diagnostic and treatment delays. The overall aim of this cross-sectional study was to understand the perceptions of newly graduated doctors (interns) toward their understanding of the lymphatic system and lymphoedema, and the extent to which both were covered within their Australian medical degree. It was found that interns perceive their understanding of the lymphatic system and lymphoedema to be suboptimal, and comprehensiveness of medical curricula specific to the lymphatic system and lymphoedema to be lacking. Findings from this research will be used to strengthen teaching of the lymphatic system and lymphoedema within Australian medical schools, thereby improving early recognition, management, and outcomes of lymphatic system disorders, such as lymphoedema.

**Abstract:**

The aim of this study was to survey the perceptions of recent (i.e., within the past 12 months) Australian medical graduates regarding (i) their understanding of the lymphatic system and lymphoedema, and (ii) the extent to which the lymphatic system and lymphoedema were covered as part of their medical degree. Medical graduates were invited to participate in a 17-item online survey that asked respondents to rate their level of agreement (using a 5-point Likert scale; higher scores = higher agreement) to statements that explored their understanding and comprehensiveness of their medical degree. Responses to each item were described using n (%). Subscale scores for understanding and medical degree were computed by summing scores of individual items, described using means (SD) and compared by participant characteristics. Medical graduates (*n* = 230) perceived their understanding of the lymphatic system and lymphoedema to be low, and comprehensiveness of medical curricula specific to the lymphatic system and lymphoedema to be lacking. Subscale scores did not differ by participant characteristics. Improvement of medical graduates understanding of lymphoedema may facilitate greater awareness of lymphoedema, thus optimizing the timeliness of diagnosis and access to treatment.

## 1. Introduction

Lymphoedema (also termed, chronic oedema) is a progressive, chronic condition that occurs when fluid accumulates in tissue spaces due to an imbalance between interstitial fluid production and the body’s ability to transport it, resulting in significant physical and psychological morbidity [1]. It may develop because of a primary congenital malformation of the lymphatic system such as Milroy disease, or as a secondary consequence of damage to the lymphatic vessels and/or lymph nodes, for example following treatment for cancer [1]. While lymphoedema has traditionally been viewed as a rare condition, lymphatic filariasis, a neglected tropical disease caused by the spread of filarial parasites by mosquitoes, has been responsible for millions of cases of lymphoedema within endemic countries for thousands of years [2]. The cause of lymphoedema in western populations is very different, with cases more often a consequence of a primary or secondary lymphatic impairment, venous insufficiency, immobility, advanced cancer, lipoedema, obesity, or vascular malformations [3].

While the prevalence of secondary lymphoedema following treatment for breast cancer is estimated to be approximately 20% [4], there has historically been a lack of population-based epidemiological studies to define its prevalence from other causes [3]. However, one recent study in the United Kingdom found that between 52 and 69% of patients cared for by community nurses had chronic oedema [5]. An Australian study found that up to 54% of patients across three health services had chronic oedema, while 100% of those presenting to a specialized wound treatment center had oedema [6]. Despite cancer-related lymphoedema arguably having received the greatest amount of research attention to date, the overwhelming majority (that is, 93%) of people with chronic oedema in this study had a non-cancer-related cause [6].

Lymphoedema is a life-long condition with no known cure. It is associated with a high burden of disease, owing to its physical, functional, psychological, and psychosocial impacts, and adversely affects quality of life [7]. Physical symptoms associated with lymphoedema include pain, heaviness, movement restriction, and changes to the skin and subcutaneous tissues including non-healing wounds and cellulitis [7,8,9], particularly in chronic advanced stages when early signs of oedema have not been recognized or managed [10]. Cellulitis, a common bacterial infection caused by Streptococcus pyogenes and/or Staphylococcus aureus, is experienced by one third of people with lymphoedema, often leading to hospitalization, worsening of underlying comorbidities, and long-term morbidity [11,12]. While cellulitis typically resolves quickly with appropriate antibiotics and may be further supported by antibiotic prophylaxis in some cases [9], recent studies have emphasized that oedema control is associated with a significantly lower risk of its development (and re-occurrence), and that measures to effectively control oedema should be mandatory [11].

While there is no cure for lymphoedema [13], correctly identifying and treating the underlying cause and/or symptoms at an early stage may reduce the incidence and severity of symptoms [14], cellulitis episodes [15], and costs to the patient and healthcare system [16]. Modern lymphatic micro-surgery is achieving global success in improving lymphoedema symptoms for patients with specific criteria [17,18]; however, it is not easily accessible for people with lymphoedema in Australia due to service availability and cost. Conservative treatment approaches are more commonly employed and recommended in the first instance even when surgery is considered (and available) [10,19]. The goal of conservative treatment is to improve oedema and associated symptoms, by using physical treatments designed to stimulate flow through either existing or collateral lymphatic pathways, such as compression garments, exercise, skin care, and manual lymphatic drainage (massage) [8]. It is commonly provided by lymphoedema practitioners, such as nurses, massage therapists, or allied health professionals, who have qualifications in lymphoedema management. However, in many cases, medical assessment, diagnosis, and subsequent medical referral is required to access these services.

Knowledge of lymphoedema among healthcare professionals, including doctors, is generally considered to be low [20], and access to doctors with the knowledge and expertise to diagnose and manage (or refer for management) lymphoedema is an established problem [21,22,23]. While the cause of this knowledge gap is unknown, some studies have suggested that it may be due to insufficient time within medical curricula allocated to the lymphatic system and lymphoedema, and failure to update curriculum frameworks in line with advancements in medical research [22,24].

In recent years, the International Lymphoedema Framework have published the Lymphoedema Education Benchmark Statements, compiled through a process of expert panel consensus as a teaching resource [25]. They were developed to reflect what a person with (or at risk of developing) chronic oedema or lymphoedema might reasonably expect from their medical or other healthcare professional, in addition to establishing global consistency in relation to lymphoedema education [25]. The benchmarks include anatomy and physiology, pathophysiology, differential diagnosis, features, educational needs of patients, and the basics of lymphoedema management [25]. The rationale for their inclusion into undergraduate curricula is due to the increasing prevalence of chronic oedema and lymphoedema, growing at-risk populations, such as the elderly, the obese, those diagnosed with cancer, and those with multiple comorbidities, as well as acknowledgment that early identification and management improves outcomes, quality of life, and costs to the healthcare system [25]. It is unclear, however, the extent to which these statements have found their way into medical curricula, if at all.

The overall aim of this study was to explore the perceptions of new medical graduates toward their understanding of the lymphatic system and lymphoedema, and the extent to which the lymphatic system and lymphoedema were encompassed within their Australian medical degree. 

## 2. Materials and Methods

An online survey was conducted with a quantitative method design conforming with the CHERRIES checklist for reporting results of internet electronic surveys (Table S1) [26]. Survey questions were developed based on the International Lymphoedema Framework Lymphoedema Education Benchmark Statements [25]. Potential eligible participants were identified from 6 June 2022–22 August 2022, through social media platforms (Facebook, Twitter, LinkedIn, Instagram) and via email to Australian universities, hospitals, clinical networks, junior doctor societies, educators, and clinicians. Social media posts and email invitations included a copy of the research flyer and survey link. Access to the participant information and consent form was provided on the survey welcome page, with online informed consent required before the survey could be commenced by clicking the required checkbox. Ethics approval was obtained from the Griffith University Human Research Ethics Committee (HREC #2022/310).

Participants were eligible to complete the survey if they had (i) completed an Australian medical degree within the past 12 months and (ii) were employed as a doctor within their first postgraduate year. Those who answered “no” to either question were not able to proceed with the survey.

The survey consisted of 17 questions that asked participants to rate their level of agreement, using a 5-point Likert scale from “strongly agree” to “strongly disagree”, to statements regarding their perceived understanding of the (i) anatomy and (ii) physiology of the lymphatic system; and (iii) pathophysiology, (iv) clinical features, (v) assessment, (vi) diagnosis, and (vii) treatment of lymphoedema. The survey then asked respondents to rate their level of agreement regarding the extent to which the (i) anatomy and (ii) physiology of the lymphatic system; and (iii) pathophysiology, (iv) clinical features, (v) assessment, (vi) diagnosis, and (vii) treatment of lymphoedema were comprehensively covered within the curricula of their Australian medical degree. The final questions asked respondents to rate their level of agreement regarding whether they perceived the amount of time devoted to (i) the lymphatic system and (ii) lymphoedema to be enough for their clinical practice, with the last question providing opportunity for open-text responses (Table S2).

All data were exported from LimeSurvey into the IBM SPSS Statistics for Windows (Version 28.0). A bot screening tool was developed and applied to remove responses that were likely derived from an internet robot (Table S3), where internet bots were defined as computer software designed to perform automated results for users and may be used by human respondents to complete surveys en masse for financial gain [27,28]. Response to items were used in two ways. First, the 16 items with 5-point Likert scale responses were collapsed into three categories: “strongly disagree or disagree”, “neutral”, and “strongly agree or agree” and *n* (%) for each of the categories were calculated and reported. Second, two subscale scores were created by summing responses to all items that explored medical graduates perceived understanding (understanding subscale, 7 items) and perceptions regarding how well the medica curricula covered the lymphatic system (medical curricula subscale, 7 items). To gauge the reliability of our survey, internal consistency of the subscale scores “understanding”, “medical curricula”, and “appropriateness for clinical practice” were calculated. To gauge the reliability of our survey, internal consistency of the subscale scores “understanding”, “medical curricula”, and “appropriateness for clinical practice” were calculated. Internal consistency of alpha (α) ≥ 0.9 was considered “Excellent”, α ≥ 0.8 as “Good”, α ≥ 0.7 “Acceptable”, α ≥ 0.6 “Questionable”, α ≥ 0.5 “Poor”, and α < 0.5 as “Unacceptable” [29]. Higher subscale scores indicated higher agreement, with the minimum score being 7 and maximum score 35. Subscale scores were described using means and standard deviations (SD). Analysis of variances were used to explore differences in the subscale scores by participant characteristics; a meaningful difference in subscale scores was a priori set at 7 units, which is indicative of a one category shift in agreement levels for each item within the subgroup. Statistical significance level was set at 0.05. Responses to open-text questions were presented descriptively and listed alongside their unique ID code.

## 3. Results

### 3.1. Respondents and Participant Characteristics

**Figure 1:**
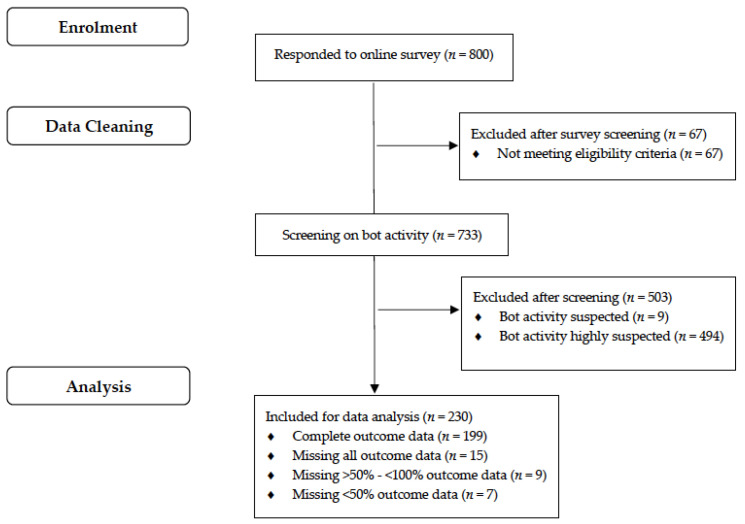
Flowchart of participant recruitment and data cleaning.

A total of 800 respondents accessed the survey. Of these, 67 did not meet eligibility criteria, while 503 respondents were identified as being potential bots (9 suspected, 494 highly suspected). Eligible respondents (*n* = 230) completed all or part of the survey and were included in subsequent analyses (Figure 1). Participant characteristics of the total eligible sample (*n* = 230) are described in Table 1. Most respondents worked in a public hospital (94.4%) within a metropolitan area (57.9%), completed a postgraduate medical degree (66.5%), and were female (57.4%). The median age of respondents was 26 years (IQR 25-28 years). Approximately one in three respondents completed their medical degree in Queensland (33.1%) and were employed in Queensland (34.4%). Participant characteristics were similar between those who provided incomplete versus complete data (Table S4). There was a higher proportion of male respondents in those categorized as “bot suspected” or “bot highly suspected” versus “no bot suspected”. A higher proportion of the undergraduate enrollment type and a lower proportion of respondents who completed their degree in Queensland, were working in Queensland, and were working in a public hospital setting were categorized as “bot highly suspected” when compared with characteristics of those categorized as “no bot suspected” or “bot suspected” (Table S5).

### 3.2. Perceptions of Understanding

**Figure 2:**
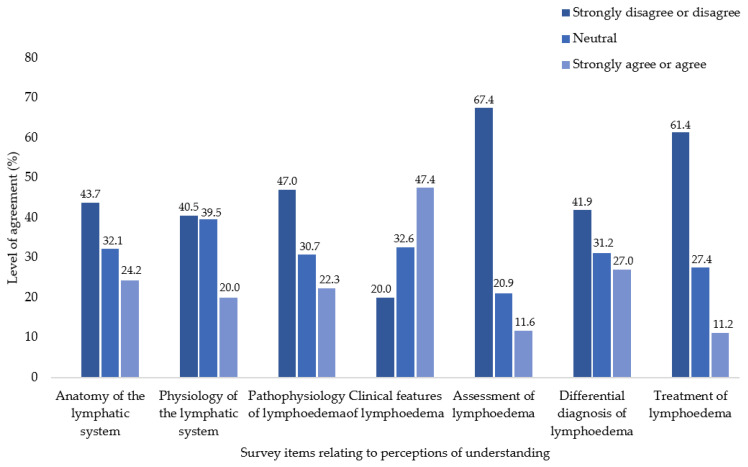
Level of agreement as to whether items listed are understood by medical graduates.

Between 40.5 and 47.0% of eligible respondents reported that they strongly disagreed or disagreed that they had a thorough understanding of the anatomy, physiology and pathophysiology of the lymphatic system, and differential diagnosis of lymphoedema (including both local and systemic causes which may co-exist), while the majority reported that they strongly disagreed or disagreed with having a thorough understanding of the methods available to assess the presence and severity of lymphoedema (67.4%) and treatment options available to manage lymphoedema (61.4%) (Figure 2). Nearly one in two (47.4%) reported to strongly agree or agree about having a thorough understanding of the clinical features of lymphoedema (Figure 2).

The reliability of the “understanding” subscale score was found to be acceptable (α = 0.77). Out of a possible score of 35 (with higher scores indicating agreement with a thorough understanding of the lymphatic system and lymphoedema), the mean and SD for the total subscale score for the sample was 18.8 (4.4) (Table 2). Agreement scores of understanding were similar, irrespective of age, enrollment type, location of medical degree, and location of clinical setting (Table 2; all *p* > 0.1). Quantitative findings were corroborated by open-text responses, such as “I don’t know much about lymphedema”, and “good recognition however very limited treatment knowledge” (Table S6).

### 3.3. Perceptions of Medical Curricula

**Figure 3:**
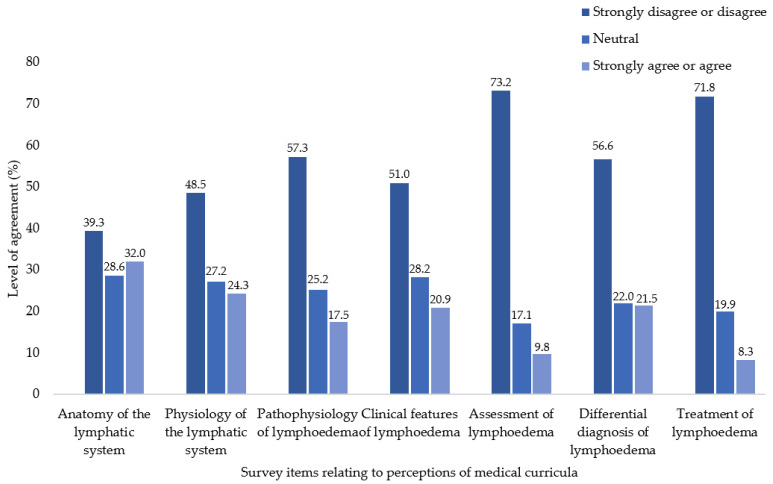
Level of agreement as to whether items listed were comprehensively covered during medical training.

The majority of respondents strongly disagreed or disagreed with five of seven statements relating to medical curricula comprehensively covering the lymphatic system and lymphoedema (Figure 3). For the remaining two items (the anatomy of the lymphatic system and physiology of the lymphatic system was comprehensively covered), 39.3% and 48.5%, respectively, strongly disagreed or disagreed. 

The reliability of the “medical curricula” subscale score was found to be good (α = 0.87). Out of a possible score of 35 (with higher scores indicating agreement with medical curricula comprehensively covering the lymphatic system and lymphoedema), the mean (SD) of medical curricula was 17.4 (5.4), and these scores were similar across respondent characteristics (Table 2; all *p* > 0.1). Open-text responses supported quantitative findings, such as “Did not hear of lymphoedema until completing medical graduate studies, only aware of symptoms and management due to my own studies during internship”, “Quite limited coverage of lymphoedema in medical school. Most of my knowledge comes from background in physiotherapy” and “This condition is not adequately covered in medical school considering how common it is to see in general practice”, but also highlighted “I think there could be slightly more teaching, but like many areas, this is quite specific post graduate knowledge. We can’t learn it all at medical school” (Table S6).

### 3.4. Perceptions of Appropriateness for Clinical Practice

The reliability of the “appropriateness for clinical practice” subscale score was found to be good (α = 0.88). Just over one in three respondents disagreed or strongly disagreed that the amount of time devoted to the lymphatic system and lymphoedema during their medical degree was appropriate for their clinical practice. Open-text responses included “Would not be able to manage lymphoedema on my own or diagnose on first presentation” and “I think I underdiagnose lymphoedema due to reduced confidence with the condition”, while others highlighted “Lymphoedema was not extensively covered but is also not something I have come across as a student or so far as a doctor” (Table S6).

## 4. Discussion

This study explored the perceptions of new medical graduates toward their understanding of the lymphatic system and lymphoedema, and the extent to which the lymphatic system and lymphoedema were encompassed within their Australian medical degree. Less than one in four participants considered their understanding of the lymphatic system to be thorough (at most 24.2%), and less than one in three participants considered the lymphatic system to be comprehensively covered (at most 32.0%) within their medical training. Similarly, for lymphoedema, only the minority of participants reported thorough understanding (at most 27.0%), with the exception of understanding clinical features (that is, symptoms) of lymphoedema (47.4%), and at most, 21.5% of participants considered lymphoedema to have been comprehensively covered within their medical training. Only one in three participants (at most 29.9%) considered the amount of time devoted to the lymphatic system and lymphoedema to be appropriate for their clinical practice. Perceptions of understanding and medical curricula were similar irrespective of sex, age, enrollment type, state of medical degree/employment, clinical setting, and location.

The obstacles that people with symptoms of lymphoedema encountered when presenting to physicians in the hope of accessing appropriate treatment were recognized in a review nearly two decades ago [24]. Apathy from physicians around lymphoedema management was highlighted, in addition to the misconception that lymphoedema was a rare disease for which there was no treatment [24]. Potential reasons for this were explored in a survey of 150 physiology chairs, with findings suggesting that lymphatic function was typically addressed within other curricula components, such as the cardiovascular system, with time devoted to the lymphatic system being less than 30 min within a typical undergraduate medical degree [24]. Vuong et al. [22] later highlighted the ongoing sparsity of the literature pertaining to lymphatic conditions and medical curricula. The authors suggested that despite the increase in understanding and research in lymphatics, it had not translated into undergraduate medical teaching; thus poor awareness and poor management were likely to continue [22]. Findings from these studies align with what was found in our survey of 230 Australian medical graduates, in that, with the exception of the clinical features of lymphoedema, doctors continue to perceive their understanding of the lymphatic system and lymphoedema to be low, and that neither were covered comprehensively within the curricula of their recently completed Australian medical degree. It therefore poses the question, how are newly graduated doctors expected to manage patients with conditions such as lymphoedema, if their training (and perhaps, that of their more senior peers) has not prepared them to do so? These findings are particularly relevant for situations where medical assessment, diagnosis, and referral by a medical doctor are required in order to gain access to treatment services, as is the case for many publicly funded lymphoedema clinics across both Australia and the United Kingdom. The results from our study must therefore be acknowledged, in that medical graduates may not have the skills required to do this. 

While an education gap has been proposed in the literature, so too has the absence of lymphology being formally recognized as a medical subspecialty, with subsequent insufficient numbers of tertiary level doctors specializing in lymphatic medicine. A 2018 study highlighted that, outside of the Netherlands and the United Kingdom, medical expertise in lymphology is not formally recognized [23]. Notably, the authors of this study identified just 10 doctors in Australia specializing in lymphology for a population of 23.5 million [23]; that is, one doctor for approximately 2.35 million people, highlighting that the demand for specialist doctors in lymphology likely well exceeds (any) availability. The absence of lymphology as a recognized medical subspecialty in itself infers that all doctors require a fundamental knowledge of the lymphatic system, as well as lymphoedema assessment, diagnosis, and management (or referral for management), underscoring the significance of the findings from this study.

Doctors are arguably the most trusted healthcare professionals when any new medical symptoms, including chronic swelling, arise. While medical doctors may not be responsible for all aspects of lymphoedema treatment, their skills in medical assessment, investigation, and diagnosis are imperative, especially where multiple comorbidities (as is often the case) may complicate the clinical picture. Furthermore, once a diagnosis has been made, doctors are well-positioned to facilitate appropriate referral to other members of the healthcare team, who are well-equipped to assist patients in managing the symptoms of their lymphatic system disorder. However, for people with lymphoedema, significant diagnostic and treatment delays have been described in the literature, with the patient perspective being that low medical awareness and availability of specialists exacerbate delays [30,31]. Consequently, there have been previous calls for medical educators to review the extent and depth of teaching pertaining to lymphatic function at a training level, to ensure that people living with lymphoedema receive a proper diagnosis and timely treatment, while ensuring that specialist clinics can offer knowledgeable care, accessible for those across both metropolitan and rural areas [30]. The results of our study firmly support these recommendations. 

The lymphatic system has been notably neglected throughout history, and its relationship to human health and disease is poorly understood [13]. Some suggest that this may be due to lymphatic conditions being “only” chronically disabling and disfiguring, when compared with diseases of the cardiovascular system, which may be fatal [32]. However, significant advances in knowledge of the lymphatic system and its associated disorders have occurred over the past several decades [13,33], with the lymphatic system now recognized as having fundamental importance to the major healthcare challenges of the 21st century including cardiovascular disease, cancer, obesity, and infection [13]. Developments have included recognition that tissue fluid balance depends critically on optimal lymphatic function, with lymphatic vessels being responsible for completing the extravascular circulation, versus traditional thinking that this was almost exclusively the result of venous reabsorption [13]. A number of causal genes responsible for the onset of primary lymphoedema have also been discovered, leading to the development of a classification algorithm to support patient management and the surveillance of known associated problems such as leukemia and congenital heart disease [34,35,36]. Impaired lymph drainage has been linked to altered fat distribution and obesity, with the reverse also suspected as being true [13]. Further, cellulitis has proven common among those with impaired lymph drainage, owing to disturbances in immune cell trafficking and compromised immunosurveillance, resulting in a global health burden and a significant number of potentially preventable hospitalizations, globally [37]. Given the significance to modern healthcare, and lack of formal specialist recognition, equipping medical graduates with a broad understanding of the lymphatic system and its associated disorders, including lymphoedema, in alignment with curricula proposed in the Lymphoedema Education Benchmark Statements, seems warranted.

While previous studies have suggested that knowledge of the lymphatic system and lymphoedema among healthcare professionals is not favorable [20], and curricula frameworks within medical degrees are deficient in these areas [22,24], this is the first study to specifically survey medical graduates about their understanding of the lymphatic system and lymphoedema, and the comprehensiveness of curricula specific to the lymphatic system and lymphoedema within their Australian medical degree. For this study, we merged survey items under subscales of (i) understanding, (ii) medical curricula, and (iii) appropriateness for clinical practice, with each demonstrating acceptable to good internal consistency [29]. The online nature of this study may be considered a strength, owing to its ability to reach medical graduates across Australia. However, it was also associated with the infiltration of bots. Bots are commonplace within internet-based research, though with the potential to evade researchers who are not aware of their presence or potential impact [28]. Bot activity was identified early in recruitment, following the promotion of the survey flyer and link on Twitter, which included details of the survey incentive (chance to win 1 of 4 $100 eGift cards). While CAPTCHA (Completely Automated Public Turing test to tell Computers and Humans Apart) verifications, designed to safeguard online surveys, were used in our study, sophisticated artificial intelligence within the current online landscape means that they may be easily bypassed [38], and a combination of automated and manual checks are recommended [39]. To remove likely bot responses, we developed a screening tool to ensure that only genuine, quality responses were included for data analysis. 

Our data represent a sample of convenience and may not be representative of the views of all recent medical graduates in Australia. The study response rate distribution across states was consistent with the expected distribution [40], but recruitment bias is likely as study advertisement was dependent on the uptake and availability of social media platforms and engagement of Australian universities, hospitals, clinical networks, junior doctor societies, educators, and clinicians. The survey also collected retrospective data, with the potential for recall bias to influence results. Given that the outcomes of interest in this study were based on respondent perception, and that respondents were not blinded to the hypothesis under investigation, the potential for participation bias is also acknowledged. However, the direction of that bias is unclear; that is, it is unknown whether those with some level of understanding of the lymphatic system were more or less likely to participate. It is conceivable that the population examined in this study (that is, medical interns in Australia) perceive their understanding and comprehensiveness of medical curricula to be deficient for systems and disorders beyond the lymphatic system and lymphoedema due to their novice status and lack of clinical experience. Findings from this study must therefore be considered in the context of this population only, with future research needed to determine whether these findings are similar among more experienced doctors and those who completed their medical training outside of Australia.

## 5. Conclusions

This study has highlighted that Australian medical graduates perceive their understanding of the lymphatic system and lymphoedema to be suboptimal and the comprehensiveness of medical curricula specific to the lymphatic system and lymphoedema to be lacking. Improvement of medical graduates understanding of lymphoedema may facilitate greater awareness of this chronic condition, thus optimizing the timeliness of diagnosis, access to treatment, symptom burden for patients, and costs to global healthcare services.

## Figures and Tables

**Table 1:** Participant characteristics of the total sample (*n* = 230).

Characteristic	*n* (%)
Sex	
Male	83 (36.1)
Female	132 (57.4)
Non-binary	3 (1.3)
Prefer not to say	5 (2.2)
Missing	7 (3.0)
Age (years); median, interquartile range (IQR)	26, 25–28
Enrolment Type	
Undergraduate	70 (30.5)
Postgraduate	153 (66.5)
Missing	7 (3.0)
State/Territory of Medical Degree	
Queensland	76 (33.1)
New South Wales	46 (20.0)
Victoria	57 (24.8)
South Australia	14 (6.1)
Western Australia	12 (5.2)
Tasmania	5 (2.2)
Australian Capital Territory	12 (5.2)
Northern Territory	1 (0.4)
Missing	7 (3.0)
State/Territory of Employment	
Queensland	79 (34.4)
New South Wales	51 (22.2)
Victoria	53 (23.0)
South Australia	14 (6.1)
Western Australia	13 (5.7)
Tasmania	3 (1.3)
Australian Capital Territory	5 (2.2)
Northern Territory	4 (1.7)
Outside Australia	1 (0.4)
Missing	7 (3.0)
Clinical Setting Type	
Public Hospital	217 (94.4)
Private Hospital	3 (1.4)
Community Health Facility	1 (0.4)
General Practice	1 (0.4)
Aged Care	0 (0.0)
Other	1 (0.4)
Missing	7 (3.0)
Clinical Setting Location	
Metropolitan area	133 (57.9)
Regional area	79 (34.4)
Rural area	10 (4.3)
Other	1 (0.4)
Missing	7 (3.0)

**Table 2:** Subscale scores (minimum: 7; maximum: 35) for perceptions related to (i) understanding of the lymphatic system and lymphoedema, and (ii) the extent to which the lymphatic system and lymphoedema were covered in medical curricula, for the total sample and by participant characteristics.

Characteristic	Understanding			Medical Curricula		
	*n*	Mean (SD)	*p*-Value	*n*	Mean (SD)	*p*-Value
Total sample (score ranging between 7–35)	215	18.8 (4.4)		205	17.4 (5.4)	
Sex			0.15			0.23
Male	79	18.9 (4.7)		75	18.1 (5.2)	
Female	131	18.9 (4.2)		126	17.2 (5.3)	
Non-binary	3	13.3 (5.5)		3	12.7 (8.1)	
Prefer not to say	2	16.0 (0.0)		1	14.0 (-)	
Age			0.79			0.15
<25	96	18.8 (4.5)		91	18.1 (5.2)	
26–30	85	18.8 (4.3)		80	17 (5.0)	
31–35	19	17.8 (5.1)		19	15.5 (7.0)	
36+	15	19.3 (3.9)		15	18.7 (5.3)	
Enrolment Type			0.76			0.37
Undergraduate	66	18.9 (4.0)		62	18 (5.8)	
Postgraduate	149	18.7 (4.6)		143	17.2 (5.1)	
State/Territory of Medical Degree			0.79			0.11
Queensland	73	19.4 (4.6)		71	18.2 (4.8)	
New South Wales	45	18.6 (4.4)		43	16.2 (5.2)	
Victoria	55	18.1 (4.7)		51	17.5 (6.1)	
South Australia	14	18.8 (3.9)		13	15 (5.1)	
Western Australia	11	18.7 (2.9)		11	20.3 (5.1)	
Tasmania	5	18.2 (4.4)		4	19 (5.8)	
Australian Capital Territory	12	18.9 (4.2)		12	16.7 (4.6)	
Northern Territory	-	-		-	-	
State/Territory of Employment			0.21			0.24
Queensland	77	19.4 (4.5)		74	18.2 (4.9)	
New South Wales	50	19.2 (4.3)		47	17.2 (5.3)	
Victoria	51	18.1 (4.2)		48	17.2 (5.8)	
South Australia	14	18.8 (3.9)		13	15.1 (5.2)	
Western Australia	12	18.0 (4.5)		12	19.3 (6.0)	
Tasmania	3	15.3 (9.1)		3	15.7 (7.6)	
Australian Capital Territory	5	18.8 (3.4)		5	16.0 (2.9)	
Northern Territory	3	13.3 (5.0)		3	12.3 (4.5)	
Outside Australia	-	-		-	-	
Clinical Setting Type			0.44			0.15
Public Hospital	210	18.7 (4.4)		200	17.5 (5.3)	
Private Hospital	3	22.3 (5.7)		3	21.7 (2.5)	
Community Health Facility	1	16.0 (-)		1	8.0 (-)	
General Practice	1	16.0 (-)		1	14.0 (-)	
Aged Care	-	-		-	-	
Other	-	-		-	-	
Clinical Setting Location			0.59			0.87
Metropolitan area	127	19.0 (4.5)		121	17.5 (5.2)	
Regional area	78	18.4 (4.5)		74	17.3 (5.6)	
Rural area	10	19.4 (3.0)		10	18.2 (5.4)	
Other	-	-		-	-	

## Data Availability

All data presented in this study is available in the article and Supplementary Materials.

## Supplementary Materials

The following supporting information can be downloaded at: https://www.mdpi.com/article/10.3390/cancers14246219/s1, Table S1: Checklist for Reporting Result of Internet E-surveys (CHERRIES); Table S2: Survey items; Table S3: Bot screening process; Table S4: Participant characteristics (*n* (%)) for those with complete and incomplete outcome data; Table S5: Participant characteristics (*n* (%)) for “no bot suspected”, “bot suspected” and “bot highly suspected” groups; Table S6: Open-text quotes from participants by topic.
