# Supplementary material for: The Lymphatic System, Lymphoedema, and Medical Curricula–Survey of Australian Medical Graduates
Source: Cancers (Basel). 2022 Dec 16;14(24):6219. doi: 10.3390/cancers14246219 (PMC9777454; doi:10.3390/cancers14246219)
Supplement: Supplementary file 1 [file cancers-14-06219-s001.zip › cancers-2034076-supplementary.pdf]

cancers

MDPI

The Lymphatic System, Lymphoedema, and Medical Curricula–Survey of Australian Medical Graduates

Natalie Kruger, Melanie L. Plinsinga, Rhian Noble-Jones, Neil Piller, Vaughan Keeley and Sandra C. Hayes

Table S1. Checklist for Reporting Result of Internet E-surveys (CHERRIES).

Item Category	Checklist Item	Explanation
Design	Describe survey design	The survey was open design, targeted toward a convenience sample of Australian medical graduates (interns), that is, those who had completed an Australian medical degree within the past 12 months and were employed as a doctor within their first postgraduate year.
IRB (Institutional Review Board) approval and informed consent process	IRB approval	Ethics approval was obtained from the Griffith University Human Research Ethics Committee (HREC #2022/310).
	Informed consent	The survey welcome page provided a concise study overview, which provided information on the (i) study aims (ii) eligibility criteria (iii) access and duration of the survey (minutes to completion) (iv) voluntary nature of participation and (v) prize draw. A link to the participant information and consent form was also provided, which included further detail on the (i) the purpose of the study (ii) investigators involved in the study (iii) participation requirements (iv) participation risks and benefits (v) use and storage of personal information (vi) how to register complaints or concerns about the study with an independent third party (Manager of Research Ethics) and (vii) privacy statement. Participants consented by acknowledging the provided information and clicking the required checkbox.
	Data protection	Demographic details and data were collected; however, they did not allow for identification verification and all survey data was non-identifiable. There was an option for participants to voluntarily provide their email address at the end of the survey to (i) go into the draw to win an eGift card and / or (ii) express interest in participating in phase two of the study. Email addresses provided were stored in a password-protected file and stored separately from survey data.
Development and pre-testing	Development and testing	The survey was developed by the study investigators and piloted with a small sample of the target population to ensure optimal language, tone and ease of completion. Technical functionality of the survey was tested by research investigators prior to dissemination.
Recruitment process and description of the sample having access to the questionnaire	Open survey versus closed survey	The survey was a combination of open and closed ended questions.
	Contact mode	Initial contact with potential respondents was made online via social media posts and e-mail.
	Advertising the survey	The survey was promoted via the investigator's networks and social media platforms (including Twitter, LinkedIn, Facebook and Instagram), including promoted advertisements to achieve maximal reach and engagement. Wording of social media posts

		was piloted with the target population to ensure optimal relevance, readability, and engagement. Further invitations to complete the survey were extended via e-mail to Australian universities, hospitals, clinical networks, junior doctor societies, educators, and clinicians.
Survey administration	Web/E-mail	The survey was administered through LimeSurvey.
	Context	LimeSurvey is a web-based survey tool securely hosted by Griffith University. Each survey built within the software has a unique access link and requires an ethics approval number prior to public access. Access links to the survey were made available via social media posts and e-mail.
	Mandatory/voluntary	Participation in the survey was voluntary.
	Incentives	Participants were given the option of providing their email address at the end of the survey, to have the chance to win 1 of 4 $100 Prezzee Smart eGift cards.
	Time/Date	Data collection occurred between 6 June 2022 – 22 August 2022.
	Randomization of items or questions	Questions were not randomised.
	Adaptive questioning	An adaptive logic was present to reduce response burden for participants.
	Number of items	Survey items were presented by group, with a minimum of 2 items per page, up to 9 items per page. A total of 17 items were presented across the survey (or 31 inc. eligibility, demographics, chance to win, up next). Questions were presented by group, (i) understanding (7 questions), (ii) medical curricula (9 questions), (iii) final question (1 open-response question).
	Number of screens	Survey items were presented by group, with a minimum of 3 screens (if eligibility criteria not met) and maximum of 8 screens (if the entire survey was completed). Progress bar feature was also enabled.
	Completeness check	All submitted responses were analysed regardless of whether the entire survey was complete or not. Participants were provided with a non-response option of 'no answer' and selection of one response option was enforced.
	Review step	Backwards navigation was enabled to allow respondents to review and/or change previously answered questions prior to final submission.
Response rate	Unique site visitor	Each submission was identified with a unique response ID code.
	View rate	View rate was not quantified.
	Participation rate	Participation rate was not quantified.
	Completion rate	All participants who were eligible and agreed to participate were provided with a unique ID response code, although not everyone who was recorded as submitting the survey responded to every question.
Preventing multiple entries from the same individual	Cookies used	Cookies were not used, to maximise participation of target group known to have access to 'hot desks' (i.e. – work desks shared with colleagues, which is common practice within Australian healthcare settings).
	IP check	IP addresses were recorded, however duplicate entries were not excluded from data analyses owing to 'hot desks'. The number of duplicate IP addresses recorded within the data included for analyses (n=230) was 22.

Analysis	Log file analysis	Raw data was screened based on our eligibility criteria. Of the included participants (n=230), open text responses were analysed to facilitate detection of duplicate entries.
	Handling of incomplete questionnaires	All eligible survey responses were included for data analyses, including participants that submitted incomplete data.
	Questionnaires submitted with an atypical timestamp	No timeframe was set as a cut-off point.
	Statistical correction	No weighting or propensity scores were placed on questions.

Table S2. Survey items.

**Participants had the following options to answer each question: Strongly Agree (5), Agree (4), Neutral (3), Disagree (2), Strongly Disagree (1), No Answer (-)**	
**Understanding**	1. I have a thorough understanding of the anatomy of the lymphatic system.
	2. I have a thorough understanding of the physiology of the lymphatic system.
	3. I have a thorough understanding of the pathophysiology of lymphoedema.
	4. I have a thorough understanding of the clinical features (inc. symptoms) of lymphoedema.
	5. I have a thorough understanding of the methods available to assess the presence and severity of lymphoedema.
	6. I have a thorough understanding of the differential diagnosis of lymphoedema (including both local and systemic causes, which may co-exist).
	7. I have a thorough understanding of the treatment options available to manage lymphoedema.
**Medical Curricula**	8. During my medical degree, the anatomy of the lymphatic system was comprehensively covered.
	9. During my medical degree, the physiology of the lymphatic system was comprehensively covered.
	10. During my medical degree, the pathophysiology of lymphoedema was comprehensively covered.
	11. During my medical degree, the clinical features (including associated symptoms) of lymphoedema were comprehensively covered.
	12. During my medical degree, the methods available to assess the presence and severity of lymphoedema were comprehensively covered.
	13. During my medical degree, the differential diagnosis of lymphoedema (including both local and systemic causes, which may co-exist) was comprehensively covered.
	14. During my medical degree, the treatment options available to manage lymphoedema were comprehensively covered.
**Clinical Practice**	15. During my medical degree, the amount of time devoted to the lymphatic system was appropriate for my clinical practice.
	16. During my medical degree, the amount of time devoted to lymphoedema was appropriate for my clinical practice.
**Final**	17. Finally, is there anything further you would like to say? <open response>

Table S3. Bot screening process.

**No bot activity suspected**
None of the below criteria are met<br>• Unusual location of traffic (outside of Australia / NZ)<br>• Unusual patterns of usage / traffic (13.6.22, 13.7.22)<br>• Nonsense response to open response question<br>• Duplicate response to open response question
**Bot activity suspected**
At least one of the below criteria are met<br>• Unusual patterns of usage / traffic (13.6.22, 13.7.22)<br>• Nonsense response to open response question<br>• Duplicate response to open response question
**Bot activity highly suspected**
• Unusual location of traffic (outside of Australia / NZ) and / or,<br>at least two of the following criteria are met<br>• Unusual patterns of usage / traffic (13.6.22, 13.7.22)<br>• Nonsense response to open response question<br>• Duplicate response to open response question

Table S4. Participant characteristics (n (%)) for the total sample and for those with complete and incomplete outcome data.

Variable	Total Sample (*N* = 230)	Complete Data (*N* = 199)	Incomplete Data (*N* = 31)
Sex			
Male	83 (36.1)	73 (36.7)	10 (32.3)
Female	132 (57.4)	122 (61.3)	10 (32.3)
Non-binary	3 (1.3)	3 (1.5)	0 (0.0)
Prefer not to say	5 (2.2)	1 (0.5)	4 (12.8)
Missing	7 (3.0)	0 (0.0)	7 (22.6)
Age (years); median, interquartile range (IQR)	26, 25-28	26, 25-28	25, 24-26
Enrolment Type			
Undergraduate	70 (30.5)	60 (30.2)	10 (32.3)
Postgraduate	153 (66.5)	139 (69.8)	14 (45.1)
Missing	7 (3.0)	0 (0.0)	7 (22.6)
State / Territory of Medical Degree			
Queensland	76 (33.1)	67 (33.7)	9 (29.1)
New South Wales	46 (20.0)	42 (21.1)	4 (12.9)
Victoria	57 (24.8)	50 (25.1)	7 (22.6)
South Australia	14 (6.1)	13 (6.5)	1 (3.2)
Western Australia	12 (5.2)	11 (5.5)	1 (3.2)
Tasmania	5 (2.2)	4 (2.0)	1 (3.2)
Australian Capital Territory	12 (5.2)	12 (6.1)	0 (0.0)
Northern Territory	1 (0.4)	0 (0.0)	1 (3.2)
Missing	7 (3.0)	0 (0.0)	7 (22.6)
State / Territory of Employment			
Queensland	79 (34.4)	71 (35.7)	8 (25.9)
New South Wales	51 (22.2)	46 (23.2)	5 (16.1)
Victoria	53 (23.0)	46 (23.1)	7 (22.6)
South Australia	14 (6.1)	13 (6.5)	1 (3.2)
Western Australia	13 (5.7)	12 (6.0)	1 (3.2)
Tasmania	3 (1.3)	3 (1.5)	0 (0.0)
Australian Capital Territory	5 (2.2)	5 (2.5)	0 (0.0)
Northern Territory	4 (1.7)	3 (1.5)	1 (3.2)
Outside Australia	1 (0.4)	0 (0.0)	1 (3.2)
Missing	7 (3.0)	0 (0.0)	7 (22.6)
Clinical Setting Type			
Public Hospital	217 (94.4)	194 (97.5)	23 (74.2)
Private Hospital	3 (1.4)	3 (1.5)	0 (0.0)
Community Health Facility	1 (0.4)	1 (0.5)	0 (0.0)
General Practice	1 (0.4)	1 (0.5)	0 (0.0)
Aged Care	0 (0.0)	0 (0.0)	0 (0.0)
Other	1 (0.4)	0 (0.0)	1 (3.2)

Missing	7 (3.0)	0 (0.0)	7 (22.6)
Clinical Setting Location			
Metropolitan area	133 (57.9)	119 (59.8)	14 (45.1)
Regional area	79 (34.4)	72 (36.2)	7 (22.6)
Rural area	10 (4.3)	8 (4.0)	2 (6.5)
Other	1 (0.4)	0 (0.0)	1 (3.2)
Missing	7 (3.0)	0 (0.0)	7 (22.6)

Table S5. Participant characteristics (n (%)) for the 'no bot suspected', 'bot suspected' and 'bot highly suspected' groups.

Variable	No Bot Suspected (*N* = 230)	Bot Suspected (*N* = 9)	Bot Highly Suspected (*N* = 494)
Sex			
Male	83 (36.1)	6 (66.7)	354 (71.7)
Female	132 (57.4)	3 (33.3)	127 (25.7)
Non-binary	3 (1.3)	0 (0.0)	9 (1.8)
Prefer not to say	5 (2.2)	0 (0.0)	1 (0.2)
Missing	7 (3.0)	0 (0.0)	3 (0.6)
Age (years); median, interquartile range (IQR)	26, 25-28	27, 25-32	28, 25-33
Enrolment Type			
Undergraduate	70 (30.5)	3 (33.3)	253 (51.2)
Postgraduate	153 (66.5)	6 (66.7)	238 (48.2)
Missing	7 (3.0)	0 (0.0)	3 (0.6)
State / Territory of Medical Degree			
Queensland	76 (33.1)	3 (33.3)	82 (16.7)
New South Wales	46 (20.0)	1 (11.1)	117 (23.7)
Victoria	57 (24.8)	0 (0.0)	85 (17.2)
South Australia	14 (6.1)	2 (22.3)	85 (17.2)
Western Australia	12 (5.2)	0 (0.0)	53 (10.7)
Tasmania	5 (2.2)	0 (0.0)	25 (5.0)
Australian Capital Territory	12 (5.2)	3 (33.3)	36 (7.3)
Northern Territory	1 (0.4)	0 (0.0)	8 (1.6)
Missing	7 (3.0)	0 (0.0)	3 (0.6)
State / Territory of Employment			
Queensland	79 (34.4)	4 (44.4)	76 (15.4)
New South Wales	51 (22.2)	1 (11.1)	88 (17.9)
Victoria	53 (23.0)	1 (11.1)	88 (17.9)
South Australia	14 (6.1)	2 (22.3)	90 (18.2)
Western Australia	13 (5.7)	0 (0.0)	60 (12.1)
Tasmania	3 (1.3)	0 (0.0)	31 (6.3)
Australian Capital Territory	5 (2.2)	1 (11.1)	29 (5.8)
Northern Territory	4 (1.7)	0 (0.0)	13 (2.6)
Outside Australia	1 (0.4)	0 (0.0)	16 (3.2)
Missing	7 (3.0)	0 (0.0)	3 (0.6)
Clinical Setting Type			
Public Hospital	217 (94.4)	8 (88.9)	236 (47.8)
Private Hospital	3 (1.4)	1 (11.1)	150 (30.4)
Community Health Facility	1 (0.4)	0 (0.0)	71 (14.4)
General Practice	1 (0.4)	0 (0.0)	23 (4.6)
Aged Care	0 (0.0)	0 (0.0)	8 (1.6)
Other	1 (0.4)	0 (0.0)	3 (0.6)
Missing	7 (3.0)	0 (0.0)	3 (0.6)

Clinical Setting Location			
Metropolitan area	133 (57.9)	5 (55.6)	276 (55.9)
Regional area	79 (34.4)	4 (44.4)	183 (37.0)
Rural area	10 (4.3)	0 (0.0)	28 (5.7)
Other	1 (0.4)	0 (0.0)	4 (0.8)
Missing	7 (3.0)	0 (0.0)	3 (0.6)

Table S6. Open-text quotes from participants by topic.

Variable	Participant ID no.	Responses
Medical training	141	Quite limited coverage of lymphoedema in medical school. Most of my knowledge comes from background in physiotherapy.
	138	It was covered in my first year of medical school and was not brought up again beyond that year so most of the knowledge was not cemented.
	816	Four year postgraduate medical degrees in Australia are notoriously shit for teaching anything useful. An overview of lymphatic anatomy was covered in the rushed initial 2 years of pre-clinical sciences but was not revisited by any formal teaching in the subsequent 2 years. I definitely never needed to learn about lymphoedema for any of my exams. In general, Australian medical degrees need to be streamlined (less extra ethics/professionalism and mandatory research activities) and more core sciences such as lymphatics/lymphoedema.
	181	Education at medical school in clinical years is deficient for ALL diseases - this problem isn't exclusive to lymphoedema. I had no idea what to study and there were no supporting lectures or readings to guide me.
	192	We had very little taught about lymphoedema, to the point that I don't know what I don't know and am confused about why research is being done on how well it's taught.
	689	Basic system was covered, as well as the basics of lymphoedema. I know about how it looks and can be treated from personal life than training.
	732	Did not hear of lymphoedema until completing medical graduate studies, only aware of symptoms and management due to my own studies during internship.
	744	I was very lucky to have a tutorial with a lymphoedema nurse and a physio main focus of this was practical demonstration of the techniques used to manage and info provided on how it affects people and statistics. However only given an article on pathophysiology to read in my own time, no formal tutorial on this from any lecturers.
	748	Anatomy of lymphedema may have been lacking during my med degree. However, pathophys, DDx and treatments were covered in detail.
	758	Anatomy and physiology didactic teaching was very fast paced/sometimes just self-directed. I suppose most of the degree was self-directed.
	783	There was one lecture about it during my clinical years.
	810	I think there could be slightly more teaching but like many areas this is quite specific post graduate knowledge We can't learn it all at medical school.

	105	I understand that lymphoedema is an important condition that can have significant negative effects for patients, however I personally wouldn't say that we did not get 'enough' teaching on this, or that there should be more teaching on this particular topic. The reality is, there are a lot of important conditions that affect our patients, and we will not be able to comprehensively understand them all in just a few years of medical school. I think what is much more important is for us to have an attitude and interest that allows us to continue to learn in our years of working as a doctor.
	759	Hard to remember.
	657	I don't know yet.
	101	We learn how to examine for enlarged lymph nodes as part of each examination e.g., head and neck, abdominal, breast exams. Then differentials for enlarged nodes were discussed - I thought that this was a very good way to learn.
Clinical practice	159	Would not be able to manage lymphoedema on my own or diagnose on first presentation.
	148	I think lymphoedema is not managed particularly well at the acute hospital where I work. I had a patient with severe lymphoedema who was known to the community lympoedema clinic but happened to have a very prolonged admission for other reasons. We wanted to get his legs properly wrapped and managed and couldn't find anyone to do it. I called the vascular CNC and wound care CNC, as well as speaking to physio and a few other departments and everyone agreed that there was no one at the hospital who especially manages it. Apparently there had been previously but she was seconded to ICU due to covid? We had to get the guy's legs wrapped by the nurses on the ward as best they could. I don't think it was the best we should have been providing for this gentleman. I see patients with chronic lympoedema often and it just seems to be 'noted' in their history and we never address it, talk about it or do anything with it while they are a patient in the acute hospital. I imagine it is different at the peripheral/rehab hospital sites but I haven't worked at one of them yet.
	184	Now that I have entered clinical practice I wish I had been given a better understanding of the clinical management of lymphedema.
	124	I think I underdiagnose lymphoedema due to reduced confidence with the condition.
Knowledge	28	I don't know much about lymphedema.
	773	The only reason I know about Lymphoedema is because I follow someone on Instagram with the condition.
	765	Good recognition however very limited treatment knowledge.
	132	Kind of a vague system with not great treatments around.
Interest	762	Through this survey I have discovered that I don't have much knowledge on lymphoedema. I would like to learn about it.
	801	Are you able to supply a little course now that I know how little I know?
	805	Would like an educational session on lymphoedema (path, dx, mx) please.

Multidisciplinary team	800	Lymphoedema nurses are a valuable resource that should definitely be highlighted and acknowledged.
	692	Lymphoedema is something I see daily! However, my knowledge is limited to 'compression'. Plus, I have no idea what actually lymphoedema actually is.
	96	This condition is not adequately covered in medical school considering how common it is to see in general practice.
	122	Lymphoedema is something I have encountered much more commonly than I expected to based on medical school teaching. Further, given the commonness of lower limb oedema and multitude of differentials, I think that regardless of the commonness of lymphoedema, distinguishing lymphoedema from other differentials should be emphasised in medical teaching.
Frequency of encounters	135	No other than this is a good topic to survey as I feel it was not taught well considering how many patients have lymphedema.
	25	Lymphoedema was not extensively covered but is also not something I have come across as a student or so far as a doctor.
	103	Although we haven't learnt about it much in medical school, I have only encountered 1 patient with this and it was a diagnosis I felt was based on exclusion of other causes for peripheral oedema.
	818	Hope this research brings a change into the existing gap in medical education re lymphatic systems.
	110	Nothing specific, would be curious to see results and know what spurred this project.
Survey	795	I think the questions in the survey are tricky to answer. Do I know enough to be able to piece together a clinical history and understand that a patient may have a chylothorax with an open thoracotomy - probably yes. Do I know enough to understand that lymphoedema is frequently encountered in the context of breast cancer and cancer surgery and that I need to seek support with this? Yes. Have I seen a patient with lower limb lymphoedema in the setting of a childhood car accident with trauma and that the presentation was related to disruption of the lymphatic channels and thus build-up of fluid in the patient's lower legs? Yes. If the survey asked me if my medical degree gave me a comprehensive overview of the anatomy, pathophysiology, and differential diagnosis of liver disease, I would probably give a more positive response because we order LFTs on 50% of our patients on every ward every day - we have to understand this. Nevertheless, my scoring would only be marginally better, because no; I don't have a comprehensive understanding of things such as autoimmune… [end of text].
Other	172	Has a friend interested in med school with lymphoedema, no satisfactory management options, questioning if med has the right approach/ for her as she has experience as patient.
